# Tuning Desolvation Kinetics with Perovskite‐Type Ion‐Conductive Modulators toward Low‐Temperature Zn Metal Batteries

**DOI:** 10.1002/advs.202524245

**Published:** 2026-01-25

**Authors:** Wenbin Wang, Xiaomin Cheng, Jing Zhang, Shuang Cheng, Zhenjiang Cao, Caiyin You, Yongzheng Zhang, Hao Li, Bixian Chen, Qinghua Guan, Yixiang Shi, Huihua Li, Qingbo Xiao, Hongzhen Lin, Jian Wang

**Affiliations:** ^1^ i‐Lab & CAS Key Laboratory of Nanophotonic Materials and Devices, Suzhou Institute of Nano‐tech and Nano‐bionics Chinese Academy of Sciences Suzhou China; ^2^ School of Materials Science and Engineering Xi’an University of Technology Xi’an China; ^3^ Helmholtz Institute Ulm (HIU) Ulm Germany; ^4^ Karlsruhe Institute of Technology (KIT) Karlsruhe Germany; ^5^ School of Textile & Clothing Nantong University Nantong China; ^6^ College of Science Nanjing Forestry University Nanjing China; ^7^ School of Electrical and Electronic Engineering Harbin University of Science and Technology Harbin China; ^8^ School of Chemistry, Engineering Research Center of Energy Storage Materials and Devices Xi’an Jiaotong University Xi’an China; ^9^ Institute of Agricultural Resources and Environment Jiangsu Academy of Agricultural Sciences Nanjing China; ^10^ School of Nano‐Tech and Nano‐Bionics University of Science and Technology of China Hefei P. R. China; ^11^ Guangdong Institute of Semiconductor Micro‐Nano Manufacturing Technology Guangdong China

**Keywords:** desolvation effect, ion‐dipole interactions, kinetic accelerator, low‐temperature operation, zinc metal battery

## Abstract

Aqueous zinc metal batteries (AZMBs) are regarded as the promising candidates for low‐cost, sustainable, but safe energy storage systems. Unfortunately, Zn metal anodes suffer from incomplete desolvation and random dendrite formation, which is attributed to sluggish diffusion kinetics resulted from the strong ion (Zn^2+^)‐dipole (H_2_O) interactions. Herein, to promote the Zn^2+^ desolvation and diffusion kinetics, the strategy of constructing perovskite‐type ion‐conductive kinetic modulators of ZnSn(OH)_6_ is initially designed and coated on the Zn metal anode (PIC‐ZSH@Zn), regulating ion behaviors against dendrite growth and side reactions of active water. As confirmed by theoretical simulations, COMSOL, time‐of‐flight second‐ionic mass spectroscopy, Raman and various electrochemical analyses, the abundant active sites synergistically weaken Zn^2+^‐H_2_O interactions to accelerate desolvation to release free Zn^2+^, effectively homogenizing the Zn^2+^ flux distribution to preferentially nucleate and plate metallic Zn. Consequently, the as‐fabricated cell maintains reversible stability of 800 h at 10 mA cm^−2^ with high Coulombic efficiency over 99% under low temperature of 0°C. The paired full cell with PIC‐ZSH@Zn presents a high‐capacity retention of nearly 80% after 1000 cycles at 1.0 A g^−1^ at 0°C, reinforcing the operation robustness of AZMBs under low temperature environments.

## Graphical abstract

A strategy of constructing perovskite‐type ion‐conductive interphase modulation layer is proposed to break down ion‐dipole interactions of [Zn(H_2_O)_x_]^2+^ to promote Zn^2+^ desolvation and diffusion kinetics against dendrite growth and active water‐induced hydrogen evolution reactions. Consequently, the cells with engineered Zn electrode exhibit long‐term duration and stabilize high‐capacity retention of nearly 80% under the low temperature of 0°C.

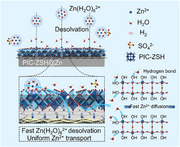

## 1 Introduction

Aqueous zinc metal batteries (AZMBs) have emerged as a promising candidate for sustainable and scalable energy storage technical solution owing to their inherent safety, cost‐effectiveness, and the abundance of zinc resources [1, 2, 3, 4, 5]. Despite their promise, Zn metal anodes face critical electrochemical instability rooted in uncontrolled dendrite formation/growth and the crack of solid electrolyte interphase (SEI) [6, 7, 8, 9]. Thereby, incomplete desolvation and cascade uneven Zn^2+^/Zn diffusion kinetics promote spatially heterogeneous deposition, ultimately limiting Coulombic efficiency (CE), cycle life, and rate stability or even triggering safety hazard in practical AZMBs [10, 11, 12, 13, 14, 15]. Besides the dendrite‐induced issues, the hydrogen evolution reaction (HER) alters local pH but also deteriorates the plating morphology, triggering irreversible Zn corrosion [16, 17, 18, 19, 20, 21], where these factors prevent the further application of AZMBs.

**FIGURE 1:**
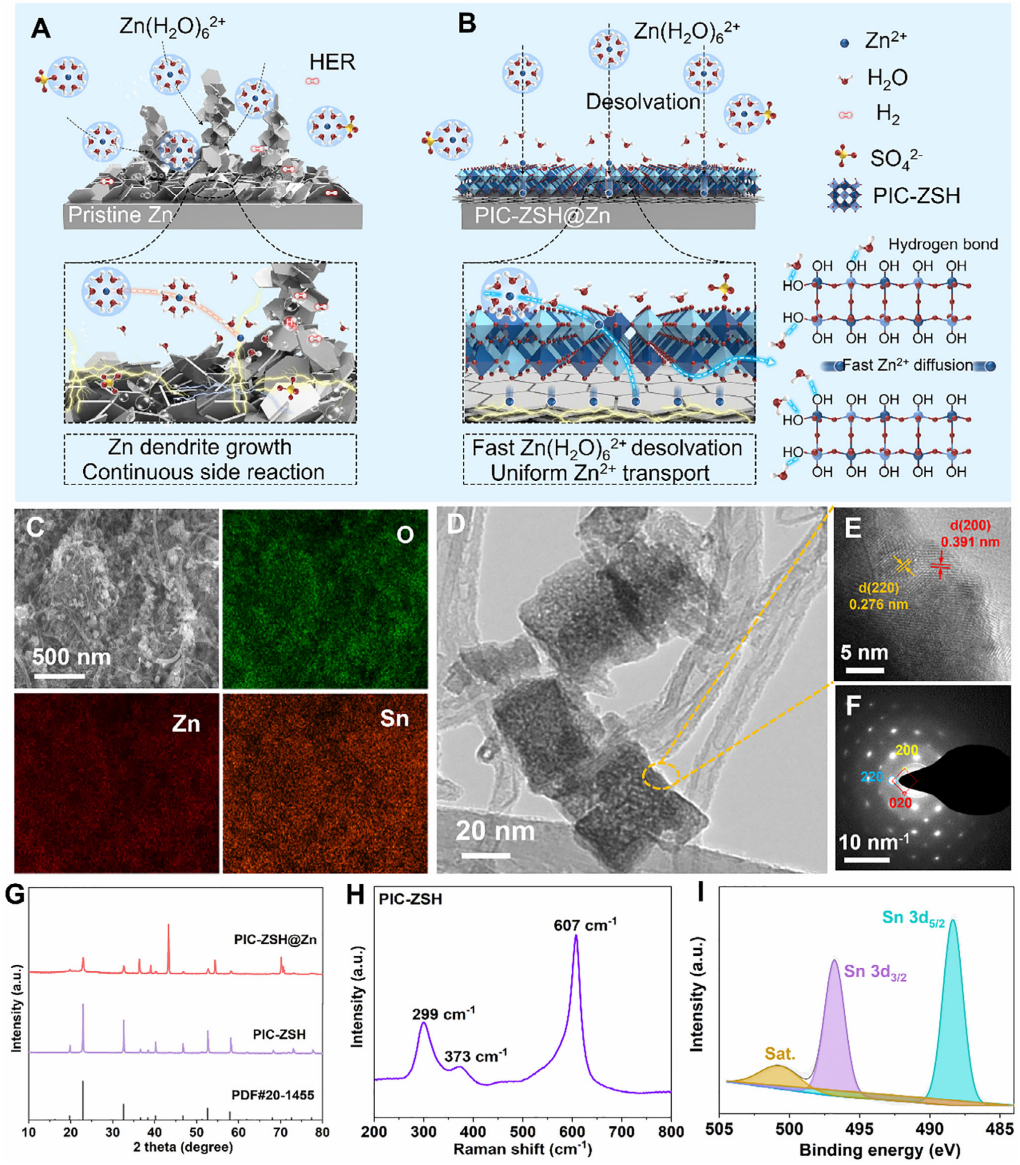
Comprehensive morphological and structural characterizations on the PIC‐ZSH nanocomposite. Schematic illustration of anode/electrolyte interface on (A) pristine Zn and (B) the PIC‐ZSH@Zn electrode with accelerated desolvation and Zn^2+^ diffusion kinetics. (C) Energy dispersive spectroscopy (EDS) elemental mappings and (D) TEM image of the PIC‐ZSH nanocomposite. (E) high‐resolution TEM image and (F) the selected area electron diffraction (SAED) image of the PIC‐ZSH nanocomposite. (G) XRD pattern of PIC‐ZSH and the PIC‐ZSH@Zn. (H) Raman spectrum of PIC‐ZSH; (I) High‐resolution XPS spectra of Sn 3d region of the PIC‐ZSH.

To solve the aforementioned issues, the targeted electrolyte engineering has emerged as rational approaches for modulating Zn^2+^ behaviors through co‐solvent additives (ethylene glycol, dimethyl carbonate) and molecular crowding solvents (polyethylene glycol, polyvinylpyrrolidone) [22, 23, 24, 25, 26, 27, 28], which competes with H_2_O for Zn^2+^ coordination or physically restricts H_2_O mobility or activity via hydrogen‐bond networks. Besides, high‐concentration electrolytes (≥3 m) also create anion‐dominated solvation structures with reduced content of H_2_O in the solvation shell structure, establishing physical barriers to enforce complete desolvation before Zn^2+^ reduction whilst homogenizing Zn^2^^+^ flux [29, 30]. Although electrolyte engineering strategies can partially modulate Zn^2+^ solvation structures, the bare Zn anodes in the aqueous acid‐like ZnSO_4_ electrolyte are still faced by incomplete desolvation‐driven sluggish Zn^2+^ diffusion, HER, and electrode corrosion due to their inability to decouple electron transfer and ion transfer, as illustrated in Figure 1A [21, 31].

Alternatively, artificial modulation interphase layers could easily establish an electron‐blocking but ion‐selective pathway, governing ion diffusion from ion‐dipole interactions [32, 33, 34, 35, 36, 37]. Nevertheless, conventional artificial coating strategies of ZnF_2_ remain inadequate in fundamentally addressing and regulating Zn^2+^/Zn interfacial transport dynamics due to their inherent high energy barriers, leading to compromised electrochemical stability and nucleation uniformity, particularly under high current densities (>5 mA cm^−2^) or sub‐ambient temperature environments (<0°C) [38, 39, 40, 41, 42]. This limitation of sluggish Zn^2+^ ion kinetics ultimately manifests in accelerated dendrite formation and capacity degradation during prolonged cycling [43, 44, 45]. It is found that Zn plating behaviors contain several steps including: [Zn(H_2_O)_6_]^2+^ migration in electrolyte; [Zn(H_2_O)_6_]^2+^ desolvation at the electrolyte/electrode interface; Zn^2+^ diffusion and Zn^0^ nucleation, where the [Zn(H_2_O)_6_]^2+^ desolvation plays a key role in the whole step. Due to the strong ion‐dipole interaction, the coordinated water molecules with central Zn^2+^ are difficult to dissociate at the pristine Zn metal surface, leaving the partially desolvated [Zn(H_2_O)_x_]^2+^ species and seriously restricting the mobility of Zn^2+^ across the SEI layer [2, 46]. Meanwhile, the strong ion‐dipole interaction would also induce the side reactions of the hydrogen evolution reaction, deteriorating the uniformity of the Zn^2+^ flux and further inducing local electric field distortion, driving Zn^2+^ to preferentially deposit (dendrite nucleation) randomly in areas [47]. Thus, it is imperative to simultaneously accelerate the interfacial desolvation kinetics of solvated Zn^2+^ complexes to establish dendrite‐free pathways for both Zn^2+^ migration and Zn atom deposition.

As is known, perovskite‐type materials, possessing various local microstructures, are beneficial for establishing 3D interconnected tunnels, which facilitates ion diffusion with enhanced ionic conductivity [48, 49, 50]. Especially, the perovskite ABX_3_ crystalline architecture stabilizes its structural framework through centrally coordinated large A‐site cations [48]. For example, perovskite‐type oxides have already been introduced in the field of battery or catalysis thanks to their bandgap tenability for charge density reconstruction, superior ionic conductivity, and intrinsic framework enabled structural stability under harsh operational conditions [51, 52]. Despite these inherent advantages for electrochemical applications, limited research efforts have been dedicated to exploring perovskite‐type materials on the interactions of ion‐dipole, especially for regulating the solvation behaviors at the interface. Therefore, taming the ion‐dipole interaction with ion‐conductive perovskite‐type compounds is beneficial for inhibiting HER and dendrite formation under the low temperature surrounding.

Herein, as a prototype, perovskite‐type ion‐conductive interphase layer of ZnSn(OH)_6_ (PIC‐ZSH) on metallic Zn electrode has initially been proposed, breaking down the interactions of ion‐dipole to promote interfacial [Zn(H_2_O)_x_]^2+^ clusters decoupling with accelerated Zn^2+^ flux for uniform deposition against dendrite growth and side reactions of HER (Figure 1B). Specially, ZnSn(OH)_6_ is endowed with rich hydroxyl (─OH) groups as coordinating ligands accompanied by the proton‐exchange‐active Sn‐OH sites, which can catalytically weaken Zn^2+^‐H_2_O interactions for expediated desolvation kinetics via adsorption and hydrogen bonding to dissociate hydration shells. Therefore, the engineered PIC‐ZSH establishes abundant active sites for synergistically accelerating the desolvation kinetics of [Zn(H_2_O)*_x_*]^2+^ groups to release free Zn^2+^ on active effect and hydrogen bond effect [53, 54], effectively homogenizing the Zn^2+^ flux distribution across the anode‐electrolyte interface to preferentially modulate inner Helmholtz plane (IHP) of the electric double layer (EDL) [13]. As determined by time‐of‐flight second‐ionic mass spectroscopy, Raman analysis, and various electrochemical measurements, as well as theoretical simulations and optical plating image evolution, the ion‐boosted interphasic layer effectively prevents interfacial electron contact from HER formation and provides high affinity to Zn^2+^ ion for fast dissociation of ion‐dipole interactions with lower energy barriers. Consequently, the PIC‐ZSH@Zn supported symmetric cells demonstrate ultralow nucleation barrier (17 mV) and extended cycling stability. Remarkably, these engineered anodes maintain dendrite‐free zinc deposition morphology even under rigorous operational conditions (10 mA cm^−2^, 0°C), showcasing superior cycling life span exceeding 800 h. Meanwhile, the full cell based on PIC‐ZSH@Zn enables high‐rate capacity of 111 mA h g^−1^ at 2 A g^−1^ as well as high‐capacity retention of 80% after 1000 cycles at 1 A g^−1^, highlighting a critical advancement toward practical AZMBs.

## 2 Results and Discussion

The typical morphology of the as‐prepared perovskite‐type ion‐conductive zinc hydroxystannate (ZnSn(OH)_6_) on the cross‐linking carbon nanotubes (CNTs) (denoted as PIC‐ZSH) accomplished by the coprecipitation method is shown in Figure 1C–F and Figures S1–S3. The scanning electron microscope (SEM) image of PIC‐ZSH (Figure S1) presents the uniform distribution of ZnSn(OH)_6_ among porous carbon nanotubes, verifying by the similar distribution of elemental Zn, Sn, O, and C on the composite (Figure 1C; Figure S2) [55]. Transmission electron microscope (TEM) images disclose the cubic‐like morphology of the as‐prepared ZnSn(OH)_6_ with the particles size around 40 nm (Figure 1D; Figure S3). The high‐resolution TEM image in Figure 1E further demonstrates the typical polycrystalline structure and the interplanar spacings of lattice fingerprint of the nanoparticle are estimated to be 0.276 and 0.391 nm, corresponding to the (220) and (200) spacing of ZnSn(OH)_6_, respectively. In the corresponding selected area electron diffraction (SAED) pattern (Figure 1F), diffraction patterns of (200), (220), and (020) are evident in the PIC‐ZSH, indicating that the high crystallinity and cubic symmetry of ZnSn(OH)_6_ is achieved. Similarly, the X‐ray diffraction (XRD) pattern presents sharp peaks centered at 22.7°, 32.4°, and 52.4°, which are assigned to the planes of (200), (220), and (420) of ZnSn(OH)_6_ (JCPDS 20–1455) phase (Figure 1G). After coating on metallic Zn surface, distinct characteristic peaks of ZnSn(OH)_6_ can also be observed in addition to the Zn peaks. Meanwhile, the PIC‐ZSH is sensitively reflected by the Raman spectrum (Figure 1H). The bands at ∼299 cm^−1^ and ∼620 cm^−1^ are assigned to the bending vibration mode of M‐Zn‐M (M representing Sn) and tensile vibration mode of M‐OH, respectively. At the same time, the spectrum in Figure S4 reveals the presence of carbon with characteristic D (∼1346 cm^−1^) and G (∼1580 cm^−1^) bands [56, 57, 58]. X‐ray photoelectron spectroscopy (XPS) was then employed to probe the surface chemical states of the PIC‐ZSH. Figure S5 indicates Zn 2p, Sn 3d, O 1s, and C 1s in the XPS spectrum, respectively. As shown in Figure S6, the high‐resolution Zn spectrum contains the two strong peaks at 1023.2 and 1046.4 eV belong to Zn 2p_3/2_ and Zn 2p_1/2_ for the Zn^2+^ state, respectively. In the high‐resolution spectrum of Sn 3d, three peaks located at around ∼488.3 eV, ∼496.7 eV, and ∼500.9 eV are assigned to Sn 3d_5/2_, Sn 3d_3/2_, and corresponding satellite peak [59, 60], respectively, signifying the presence of Sn^4+^ in the ZnSn(OH)_6_ (Figure 1I). Moreover, the features of metal hydroxide and heterostructure are disclosed in the high‐resolution O 1s and C 1s spectra (Figure S6).

**FIGURE 2:**
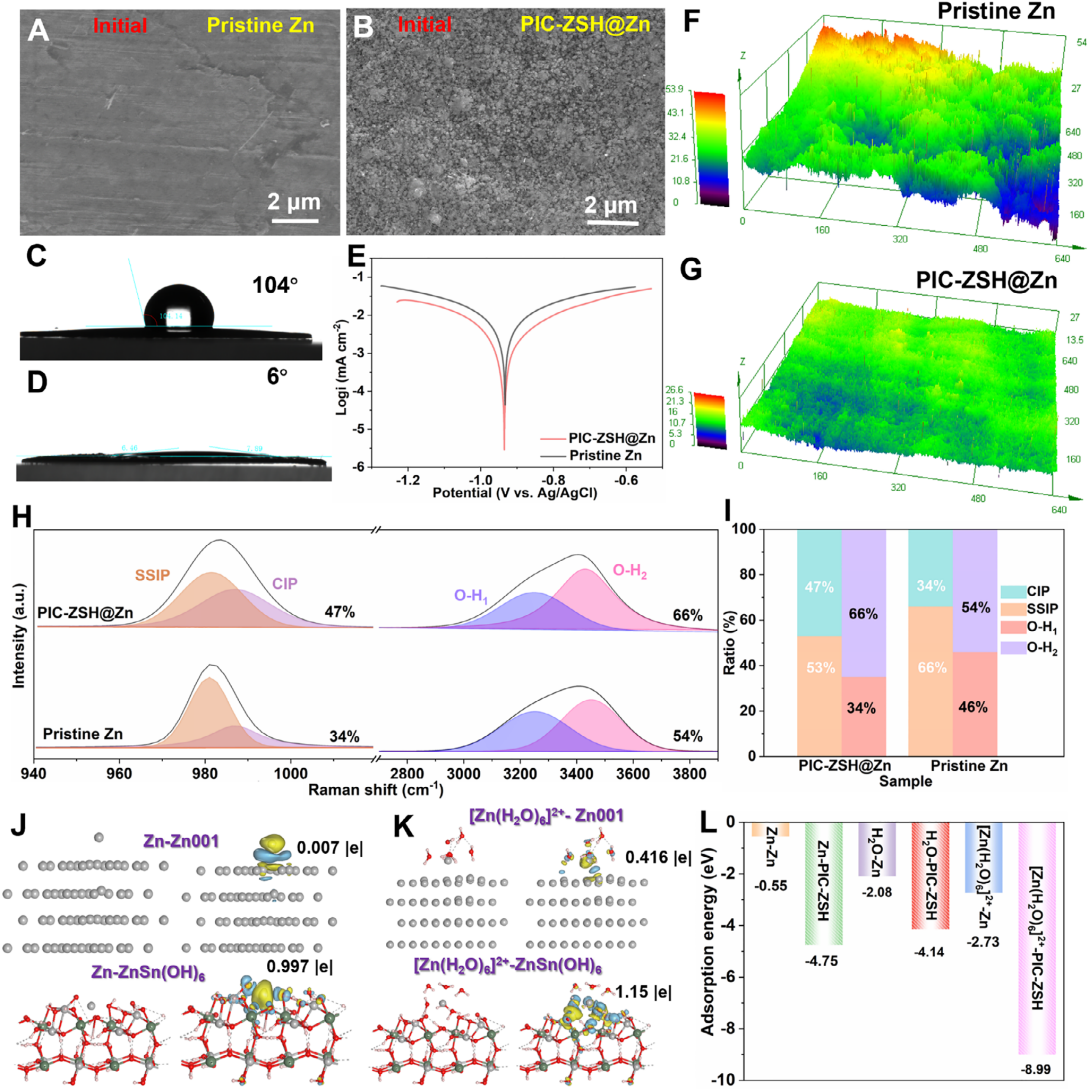
Ion‐dipolar PIC‐ZSH layer on removing active water molecules and side reactions. SEM image of (A) bare Zn and (B) PIC‐ZSH@Zn electrode. Comparison of contact angles of the electrolyte on the (C) bare Zn and (D) PIC‐ZSH@Zn. (E) Tafel plots of pristine Zn and PIC‐ZSH@Zn on a three electrodes system. Confocal microscopic 3D morphology of the immersed (F) pristine Zn and (G) PIC‐ZSH@Zn after removing the upper modulation layer. (H) Raman spectra of *v*‐SO_4_^2−^ and O‐H bonds collected on different electrode/electrolyte interfaces and (I) the corresponding ratio summary in different test systems. The adsorption configurations of (J) Zn atom on and (K) [Zn(H_2_O)_6_]^2+^ groups on pristine Zn and PIC‐ZSH@Zn, and their corresponding charge density difference upon adsorption. (L) Comparison of adsorption energy of Zn atoms, active H_2_O, and [Zn(H_2_O)_x_]^2+^ groups on pristine Zn and the PIC‐ZSH@Zn surfaces, respectively.

As shown in Figure 2A,B and Figure S7, in comparison to the relatively smooth surface of Zn metal, the ZnSn(OH)_6_ layer was coated on the pre‐polished Zn metal surface. As expected, the PIC‐ZSH modulated Zn in Figure 2C,D presents super‐hydrophilicity superior to the almost hydrophobic pristine Zn (6° vs. 104°), forming more hydrophilic surface for wetting. The enhanced interfacial wettability is bound to reduce the electrochemical impedance arising from the interaction between reactive water molecules and the Zn metal surface [61]. Such optimization might benefit the expulsion of free water molecules from the solvation sheath. The formed hydrogen bond between [Zn(H_2_O)_6_]^2+^ and PIC‐ZSH would promote the breaking of ion‐dipole interactions to accelerate the step‐by‐step dissociation of the solvated sheath layer with decreased energy barrier. Thereby, the hydrophilicity of the interface is closely related to the desolvation kinetics of zinc ions by optimizing the hydrogen bond rearrangement path and charge transfer channel. At the same time, the PIC‐ZSH layer not only diminishes diffusion steric hindrance to minimize the barriers for Zn^2+^ migration but also suppresses HER by mitigating water‐induced parasitic side reactions at the electrode‐electrolyte interface. Tafel polarization curve was conducted on the PIC‐ZSH@Zn electrode to validate the anticorrosion performance, revealing a significant suppression of corrosion current density from 5.1x10^−3^ to 8.2x10^−4^ mA cm^−2^ for the PIC‐ZSH@Zn (Figure 2E) [62]. This decrease in corrosion current density demonstrates enhanced interfacial desolvation and stability through effective inhibition of HER. Moreover, under natural immersion for three weeks, the Zn electrode protected by the PIC‐ZSH stands smooth and uniform surface morphology (Figure 2F,G; Figures S8 and S9), while the pristine Zn counterpart develops rough surface with pronounced corrosive degradation under same immersion condition (Figure 2F; Figure S9).

During the desolvation process, the interfacial transformation at the electrode/electrolyte interface can be reflected by the accelerated dissociation kinetics for speeding up ion‐dipole interactions in [Zn(H_2_O)_6_]^2+^ complex. Evidencing by spectroscopic Raman shifts [63, 64], solvent‐separated ion pairs (SSIPs, [Zn^2+^(H_2_O)_6_·SO_4_^2−^]), where sulfate anions remain indirectly coordinated via solvent‐bridged interactions, and contact ion pairs (CIPs, [Zn^2+^(H_2_O)_5_·SO_4_^2−^]), involving direct anion coordination through partial displacement of hydration‐shell water molecules, were observed. Deconvolution of the *v*‐SO_4_^2−^ symmetric stretching and O‐H vibrational signatures are separated to underscore the desolvation efficiency and interfacial ion‐dipole dissociation mechanisms. As verified in Figure 2H, the proportion of CIPs exhibits a progressive increase with the PIC‐ZSH, demonstrating a pronounced enhancement in the coupling interaction between Zn^2+^ and SO_4_^2+^ ions assisted by the dissociation of ion‐dipolar Zn^2+^ and active water molecules. Notably, the PIC‐ZSH@Zn/electrolyte interface achieves 47% of CIPs, surpassing 34% of that observed at the pristine Zn/electrolyte interface (Figure 2I). The O─H stretching vibrations (3000–3800 cm^−1^) can be resolved into two distinct components: a stronger hydrogen‐bonding interactions (O‐H_1_) and a weaker hydrogen‐bonding configurations (O‐H_2_), which reflects the bonding environment of active water molecules within the system (Figure 2H) [40]. The PIC‐ZSH@Zn/electrolyte interface demonstrates a diminished proportion of *v*‐OH_1_ configurations (53% vs. 66%) and smaller area ratio of *v*‐OH_2_ band (15.5% vs. 30.5%) compared to the untreated Zn (Figure 2I; Figure S10), directly corroborating the role of the PIC‐ZSH interface in restructuring of hydrated Zn^2+^ solvation shells and weakening the water interactions to promote interfacial Zn^2+^ kinetics through modulating the corresponding ion‐dipole interactions. Furthermore, the density functional theory (DFT) simulation of adsorption energy on the PIC‐ZSH modulator has been simulated. As shown in Figure 2J, charge differential density (CDD) simulation reveals a charge transfer of 0.997 e^−^ between PIC‐ZSH and Zn atoms, significantly exceeding that on bare Zn (0.007 e^−^). This pronounced enhancement is likely attributed to abundant proton‐exchange‐active Sn‐OH sites within PIC‐ZSH and hydrogen bonding effects, which collectively promote rapid charge transfer kinetics for fast desolvation of [Zn(H_2_O)_x_]^2+^ groups (Figure 2K). As expected, the PIC‐ZSH@Zn electrode presents higher adsorption energy (8.99 vs. 2.73 eV) to [Zn(H_2_O)_x_]^2+^ groups than pristine Zn electrode due to the hydrogen‐bond effect (Figure 2L). This interaction provides strong modulation capability in the adsorption of solvated Zn^2+^ for easily breaking up Zn^2+^‐H_2_O interactions to assist the following desolvation process, thereby, a lower Ea for PIC‐ZSH (37.1 vs. 64.5 kJ mol^−1^) directly quantifies its reduced barrier to enhance desolvation kinetics at low temperatures (Figure S11).

**FIGURE 3:**
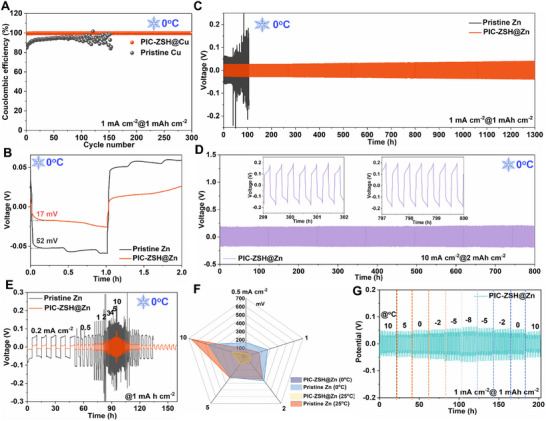
Dendrite‐free plating behavior under low temperature enabled by PIC‐ZSH engineered Zn electrode. (A) Comparison of CE on PIC‐ZSH@Cu and pristine Cu. (B) Initial nucleation voltage on the PIC‐ZSH@Zn and pristine Zn electrodes at 1 mA cm^−2^ under 0°C. Galvanostatic Zn stripping/plating behavior (C) at 1 mA cm^−2^ and (D) 10 mA cm^−2^ under the low temperature of 0°C. (E) Galvanostatic Zn stripping/plating from 0.2 to 10 mA cm^−2^ at 0°C. (F) Comparison of overpotential distribution on the PIC‐ZSH@Zn and pristine electrodes with the increase of current density under room and low temperatures. (G) Plating/striping performance of the electrodes at 1 mA cm^−2^ with 1 mA h cm^−2^ under a varying temperature from 0°C to −8°C.

Coulombic efficiency (CE) can effectively assess the utilization efficiency of the plated Zn [4, 65, 66]. Given that the PIC‐ZSH coating facilitates Zn^2+^ desolvation/diffusion kinetics and suppresses dendritic growth through regulating the ion‐dipole interactions, the electrochemical performance of asymmetric PIC‐ZSH@Cu||PIC‐ZSH@Zn cells were investigated. Under a current density of 1 mA cm^−2^, the PIC‐ZSH‐modified cell demonstrates stable CE around ∼99.8% over 300 cycles at low temperature (Figure 3A). In contrast, the cell employed with pristine Zn exhibits significantly lower CE value with rapid degradation and premature failure within 150 cycles. Meanwhile, the voltage profile of the PIC‐ZSH@Zn electrode exhibits a smooth deposition/stripping plateau with an initial overpotential of 100 mV, which progressively stabilizes at ∼50 mV after 300 cycles (Figure S12), demonstrating enhanced interfacial reversibility. This notable reduction in polarization, coupled with sustained high CE (>99.8%), confirms the robust tolerance of the PIC‐ZSH under harsh electrodeposition conditions. Electrochemical impedance spectroscopy (EIS) was performed on symmetric cells to elucidate the optimized interfacial ion/electron kinetics of PIC‐ZSH (Figure S13). The PIC‐ZSH@Zn electrode demonstrates a remarkable reduction in charge‐transfer resistance (85 vs. 200 Ω), which is consistent with the lower contact angle for better wettability optimization and corrosion suppression.

The plating/stripping stability was assessed on PIC‐ZSH@Zn||PIC‐ZSH@Zn and Zn||Zn symmetric cells. Initially, the nucleation barrier in the PIC‐ZSH@Zn electrode at low temperature of 0°C is remarkably decreased from 52 mV of pristine Zn to 17 mV (Figure 3B). In the following cycling, the Zn||Zn cell exhibits an overpotential exceeding 300 mV after 100 h, followed by pronounced fluctuations at 1 mA cm^−2^. In contrast, the PIC‐ZSH@Zn||PIC‐ZSH@Zn cell demonstrates greater stability over 1300 h with ultra‐low average overpotential of 70 mV (Figure 3C), highlighting the enhanced plating/stripping lifespan with PIC‐ZSH modification. Also, simultaneously increasing current density and areal capacity to 10 mA cm^−2^ and 2 mAh cm^−2^, the PIC‐ZSH@Zn electrode maintains smooth overpotential as low as 400 mV over extended durations of 800 h (Figure 3D). Even rising the deposition capacity to ultra‐high capacity of 10 mA h cm^−2^, the PIC‐ZSH@Zn electrode can maintain consistent performance less than 200 mV for over 110 h under high current density of 5 mA cm^−2^ (Figure S14), demonstrating exceptional tolerance to modulate the ion‐dipole interactions. Switching the current density to evaluate the rate performance, the PIC‐ZSH@Zn electrode demonstrates stable cycling with flat overpotential across a range of current densities from 0.2 to 10 mA cm^−2^ (Figure 3E). Even reaching high‐current operation at 10 mA cm^−2^, the cell still exhibits a minimal overpotential of ∼180 mV, in comparison to the fluctuant overpotential as high as 450 mV of pristine Zn. In response to the current density to 0.5 mA cm^−2^, the overpotential of the PIC‐ZSH@Zn electrode returns to initial value (33 vs. 35 mV). This exceptional reversibility and rate stability are also disclosed under the room temperature condition, implying excellent temperature robustness of the PIC‐ZSH@Zn electrode (Figure 3F). With the help of the improved solidification and temperature tolerance of ion conductivity for the 2 m ZnSO_4_ electrolyte (Figure S15 and S16 and Table S1), the PIC‐ZSH@Zn electrode demonstrates exceptional temperature tolerance to maintain stable electrochemical performance even under extreme low‐temperature conditions of −8°C (Figure 3G). Notably, it exhibits a stable overpotential increase no more than130 mV whilst effectively suppressing zinc dendrite formation, showcasing enduring plating/stripping behavior across wide operation temperature. Under the room temperature, the PIC‐ZSH@Zn electrode achieves ultralow overpotential of 70 mV during 900 h cycling at 1 mA cm^−2^ (Figure S17A). Even increasing to high current density of 3 mA cm^−2^, steady plating/stripping with negligible voltage fluctuation (<3% deviation) is remained (Figure S17B). Under identical operational conditions, pristine Zn electrode exhibits rapid failure merely within 500 h. Shifting the current density from 0.2 to 10 mA cm^−2^, the PIC‐ZSH@Zn electrode stills maintain impressive potential stability (Figure S18).

**FIGURE 4:**
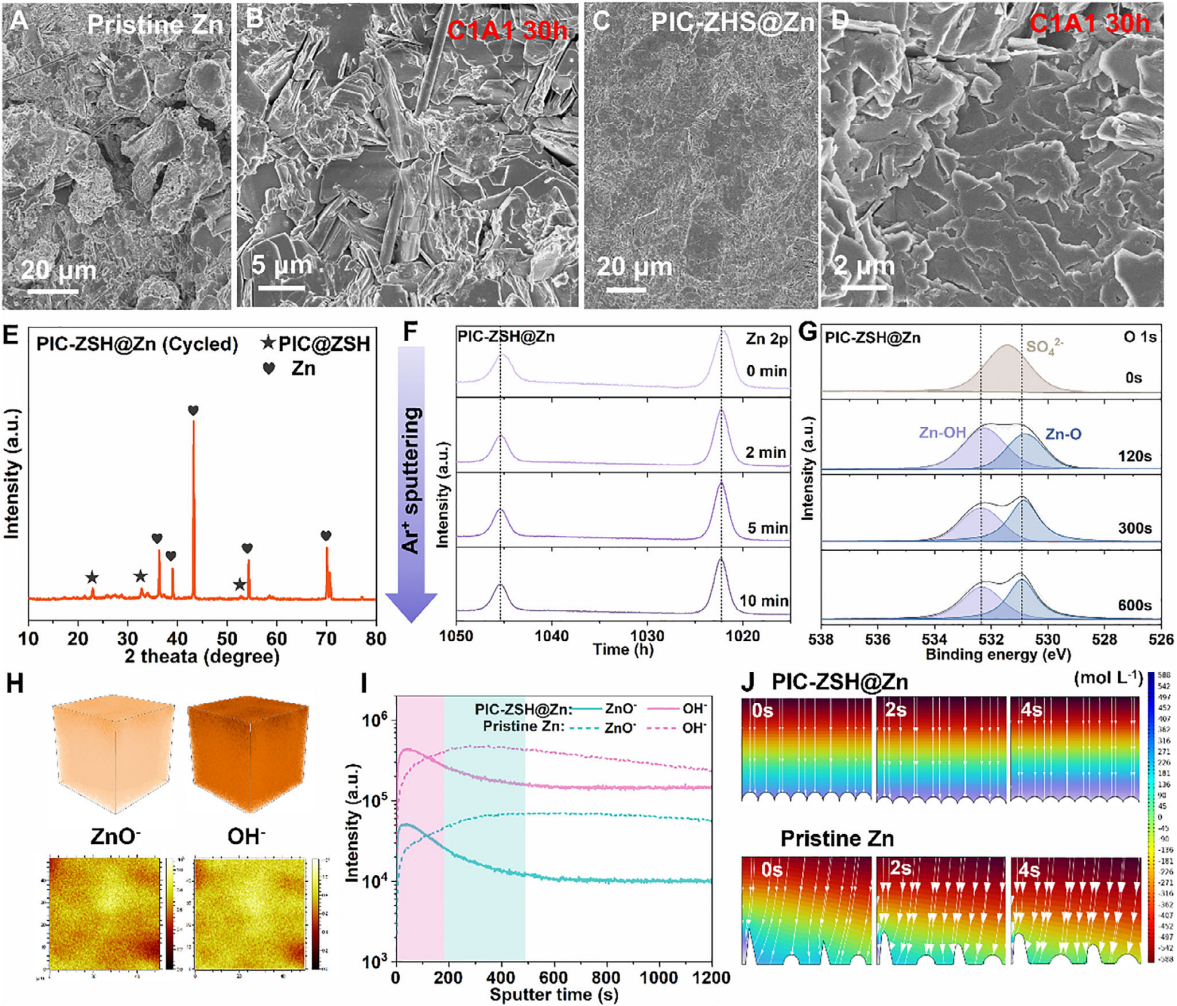
Mechanism of PIC‐ZSH ion‐dipolar modulator in accelerating Zn^2+^ desolvation for lateral Zn electroplating. SEM morphology of the cycled (A‐B) pristine Zn and (C‐D) PIC‐ZSH@Zn electrodes. (E) XRD pattern of the PIC‐ZSH@Zn electrode after cycling for 30 h. XPS depth profiles of SEI formed on the Zn/electrolytes interface after cycling for 30 h at 0°C, including high‐resolution (F) Zn 2p spectra and (G) O 1s spectra for PIC‐ZSH@Zn electrode. (H) Interfacial 3D and 2D reconstruction of species within interfacial SEI on PIC‐ZSH@Zn. (I) Intensity comparison of species within SEI on cycled PIC‐ZSH@Zn and pristine Zn electrodes. (J) COMSOL simulation of the Zn^2+^ flux distributions on the PIC‐ZSH@Zn and pristine Zn electrodes from a side view, respectively.

As evidenced by SEM observation, the annoyed vertical dendrite propagation accompanied by severe cracks are observed on pristine Zn electrode (Figure 4A,B). In contrast, the bottom‐up deposition modality shows fundamentally difference. The cycled PIC‐ZSH@Zn electrode preserves ultra‐dense zinc deposition morphology without any dendrite or corrosion substances under 0°C (Figure 4C,D), which is confirmed by XRD of the cycled PIC‐ZSH@Zn electrode (Figure 4E). As shown in Figure S19, randomly edge‐localized Zn clusters appear only within 20 min of deposition. These protrusions progressively evolve into dendritic structures over time, exhibiting pronounced tip‐driven growth by 60 min. In contrast, the PIC‐ZSH@Zn anode maintains a dendrite‐free and densely packed surface throughout the process, demonstrating the regulation of rapid desolvation and accelerated interfacial Zn^2+^ diffusion kinetics on homogeneous Zn deposition under stable ZnSn(OH)_6_ layer. In this regard, the unique zincophilicity of ZnSn(OH)_6_ greatly facilitates the dissociation of ion‐dipolar Zn^2+^‐H_2_O interactions while effectively stripping away water molecules and inhibiting anion interference, thereby releasing abundant free Zn^2+^ to enable homogeneous electrodeposition and inhibiting side reactions simultaneously. To capture the surface chemical evolution on the surface, Ar^+^‐etched in‐depth XPS was systematically performed, as shown in Figure 4F,G and Figure S20. As etching time goes on, the peak intensities of Sn 3d species in the PIC‐ZSH layer fade gradually (Figure S20). Meanwhile, across different etching depths, both the PIC‐ZSH@Zn and pristine Zn electrodes present characteristic spin‐orbit splitting in the Zn 2p spectra with dual peaks corresponding to Zn 2p_1/2_ (∼1045.3 eV) and Zn 2p_3/2_ (∼1022.2 eV) states (Figure 4F; Figure S20), respectively. After etching, a positive shift to higher binding energy confirms valence state reduction from Zn^2+^ to metallic Zn^0^ due to the existence of PIC‐ZSH modulation layer and the formation of an inert protective layer of ZnO. Simultaneously, the emergence of ZnO signature (∼530.7 eV) in O 1s spectra for both electrodes demonstrates an electrochemically inert ZnO phase forming on the electrode surface that structurally suppresses parasitic reactions and dendrite nucleation (Figure 4G; Figure S20).

Subsequently, 3D and 2D reconstructions are determined by time of‐flight secondary‐ion mass spectrometry (TOF‐SIMS) to detect the status of solid electrolyte interphase (SEI) layer and plating morphology, as displayed in Figure 4H and Figure S21. In comparison to a bulky layer of apparent rupture Zn with rough plating surface of the pristine Zn (Figure S21), the PIC‐ZSH artificial solid electrolyte interphase (SEI) layer completely suppresses both dendritic protrusions and fracture development throughout repeated electrochemical plating/stripping (Figure 4H), even subjected to elevated current densities up to 1 mA cm^−2^ for 30 h cycling under low temperature of 0°C. Under ion sputtering, the Zn‐related species (such as ZnO^−^) along with solvent‐derived species (notably OH^−^) on the zinc electrode surface exhibit an initial increase followed by a gradual decline before stabilizing at constant levels with prolonged sputtering time (Figure 4I). Moreover, the prolonged SEI evolution duration is observed in pristine Zn compared to PIC‐ZSH‐regulated Zn, which reveals the interfacial instability assigning to thicker SEI layer formation on cycled pristine Zn, ultimately allowing progressive accumulation of electrolyte decomposition and reaction products at the electrode‐electrolyte interface (Figure 4I). As illustrated in Figure 4J and Figure S22, the PIC‐ZSH modulation enables a homogeneous Zn^2+^ distribution during electroplating processes with negligible interfacial concentration gradient at the electrolyte/electrode interface. This homogeneous Zn^2+^ distribution arises from the optimized local ion pathway for transport under PIC‐ZSH regulation. However, the pristine Zn interface depicted a dendrite latency attributed to extremely uneven local electric and ion flux (Figure 4J; Figure S22). Overall, the inherent zincophilic PIC‐ZSH layer can provide fast Zn^2+^ desolvation to enable smooth ion transport, effectively regulating the deposition of Zn^2+^ and inhibit the dendrites growth to obtain smooth and dense deposition surface.

**FIGURE 5:**
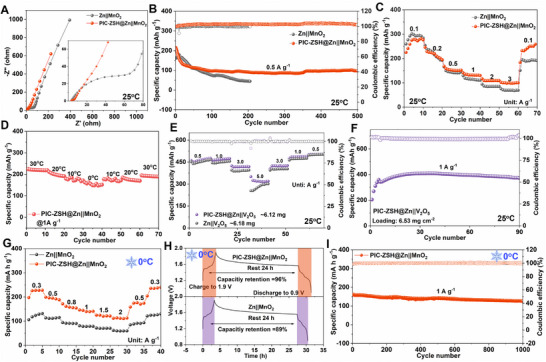
Electrochemical performance of full batteries supported by PIC‐ZSH@Zn anode. (A) EIS spectra for full cells based on PIC‐ZSH@Zn and pristine Zn anodes. (B) Long‐term cycle performance of full cells under room temperature. (D) Cycling performance of PIC‐ZSH@Zn||MnO_2_ full cell at 1 A g^−1^ under shifting temperature; (E) Rate and (F) cycling performance of the PIC‐ZSH@Zn||V_2_O_5_ full cells with high mass loading under room temperature. Rate performance of the two full cells under (G) room temperature and (H) low temperature of 0°C. (I) Self‐discharge behavior of full cells under resting for 24 h. (F) Comparison of long‐term cycle stability of the PIC‐ZSH@Zn||MnO_2_ full cell under low temperature of 0°C.

In evaluating the practical performance of the PIC‐ZSH@Zn anode, the full cell with MnO_2_ cathode demonstrates the reduced charge transfer resistance (5 vs. 80 Ω) and significantly enhanced ion diffusion kinetics compared to the pristine Zn one (Figure 5A). Consequently, the PIC‐ZSH modified full cell displays exceptional cyclability, which maintains 98 mAh g^−1^ after 500 cycles at 0.5 A g^−1^ (Figure 5B). The Mn dissolution would be responsible for the decreased capacity in the first 50 cycles. In stark contrast, the pristine Zn counterpart suffers catastrophic degradation, plummeting to a mere 45 mAh g^−1^ after 200 cycles. Figure 5C demonstrates that the PIC‐ZSH modified full cell delivers a high specific capacity of 279 mAh g^−1^ at 0.1 A g^−1^ and retains 100 mA h g^−1^ at an elevated rate of 3 A g^−1^. This remarkable capacity retention strongly validates the exceptional structural stability of the PIC‐ZSH in modulating ion‐dipole interactions. Also, the temperature robustness of the PIC‐ZSH@Zn||MnO_2_ full cell has been verified. The discharge capacity stepwise decreases from 224 mAh g^−1^ at 30°C to 162 mAh g^−1^ under 0°C with robust discharge plateaus (Figure 5D; Figure S23), confirming structural stability of PIC‐ZSH@Zn electrode under low‐temperature operation. As shown in Figure 5E,F, the PIC‐ZSH@Zn electrode coupled with high‐loading V_2_O_5_ (∼6.12 mg cm^−2^) cathode exhibits ultra‐steady rate capacity at shifting current density and an excellent capacity retention of nearly ∼100% (∼6.53 mg cm^−2^), which is comparable to the reported zinc ion battery systems (Table S2). Decreasing to low‐temperature conditions (0°C), the PIC‐ZSH@Zn‐based full cell also delivers 227 mA h g^−1^ at 0.3 A g^−1^ and retains 111 mA h g^−1^ at a high current density of 2 A g^−1^ (Figure 5G). However, the pristine Zn||MnO_2_ counterpart collapses to a mere 62 mA h g^−1^ under 2 A g^−1^, only about half capacity in that cell with the PIC‐ZSH modification. Further, resting for 24 h, the PIC‐ZSH@Zn||MnO_2_ cell keeps the capacity retention of 96% of its initial capacity (Figure 5H), while the untreated Zn||MnO_2_ only offers the capacity of 89% under identical conditions. Remarkably, the PIC‐ZSH@Zn anode enables a high initial capacity of 193 mAh g^−1^ and stabilizes at 160 mAh g^−1^ with high‐capacity retention near 80% over 1000 cycles at 1 A g^−1^ (Figure 5I), attributing to the improvement in ion diffusion kinetics for extreme‐environment energy storage applications. After cycling, the MnO_2_ cathode discloses the integrated morphology, resembling the original one without the formation of any by‐products (Figures S24 and S25) as indicated by SEM and XRD.

## 3 Conclusions

In summary, the innovative solvation structure modulation strategy is successfully achieved in reforming Zn deposition surface, where the zincophilic perovskite‐type ion‐conductive ZnSn(OH)_6_ is constructed as ion diffusion regulator. The as‐prepared PIC‐ZSH interphase achieves zinc plating stabilization against dendrite growth, side reactions of HER, and corrosion through fast desolvation and diffusion kinetics. Consequently, this synergistic active effect yields the high Coulombic efficiency exceeding 99% at 1 mA cm^−2^, extending the lifespan above 1300 h at 1 mA cm^−2^ with dendrite‐free deposition morphology, and maintaining reversible stability of 800 h at 10 mA cm^−2^ under low temperature of 0°C. The paired PIC‐ZSH@Zn||MnO_2_ full cell presents a high‐capacity retention of nearly 80% after 1000 cycles at 1.0 A g^−1^ in cryogenic environment of 0°C, underlining the viability in extreme‐environment energy storage applications for AZMBs.

## Conflicts of Interest

The authors declare no conflicts of interest.

## Supporting information




**Supporting File**: advs74075‐sup‐0001‐SuppMat.docx.

## Data Availability

The data that support the findings of this study are available from the corresponding author upon reasonable request.
