# Supplementary material for: Tuning Desolvation Kinetics with Perovskite‐Type Ion‐Conductive Modulators toward Low‐Temperature Zn Metal Batteries
Source: Adv Sci (Weinh). 2026 Jan 25;13(19):e24245. doi: 10.1002/advs.202524245 (PMC13045423; doi:10.1002/advs.202524245)
Supplement: Supplementary file 1 — Supporting File: advs74075‐sup‐0001‐SuppMat.docx. [file ADVS-13-e24245-s001.docx]

**Supporting information**

**Tuning Desolvation Kinetics with Perovskite-type Ion-conductive Modulators Toward Low-Temperature Zn Metal Batteries**

*Wenbin Wang, Xiaomin Cheng, Jing Zhang*, Shuang Cheng, Zhenjiang Cao, Caiyin You, Yongzheng Zhang, Hao Li, Bixian Chen, Qinghua Guan, Yixiang Shi, Huihua Li*, Qingbo Xiao, Hongzhen Lin*, Jian Wang**

W. Wang, X. Cheng, S. Cheng, B. Chen, Q. Guan, H. Lin

*i*-Lab & CAS Key Laboratory of Nanophotonic Materials and Devices, Suzhou Institute of Nano-tech and Nano-bionics, Chinese Academy of Sciences, Suzhou 215123, China

E-mail: hzlin2010@sinano.ac.cn; wangjian2014@sinano.ac.cn

C. You, J. Zhang

School of Materials Science and Engineering, Xi’an University of Technology, Xi’an 710048, China

E-mail: zhangjing2020@xaut.edu.cn

H. Li, J. Wang

Helmholtz Institute Ulm (HIU), Ulm D89081, Germany; Karlsruhe Institute of Technology (KIT), Karlsruhe D76021, Germany

E-mail: jian.wang@kit.edu

Y. Zhang

School of Textile & Clothing Nantong University, Nantong 226019, China

Y. Shi

College of Science, Nanjing Forestry University, Nanjing 210037, China

H. Li

School of Electrical and Electronic Engineering, Harbin University of Science and Technology, Harbin 150080, P. R. China

E-mail: li.huihua@hrust.edu.cn

Z. Cao

School of Chemistry, Engineering Research Center of Energy Storage Materials and Devices, Xi’an Jiaotong University, Xi'an 710049, China

Q. Xiao

Institute of Agricultural Resources and Environment, Jiangsu Academy of Agricultural Sciences, Nanjing 210014, China.

W. Wang, S. Cheng, Q. Guan, H. Lin

School of Nano-Tech and Nano-Bionics, University of Science and Technology of China, Hefei 230026, China

X. Cheng

Guangdong Institute of Semiconductor Micro-Nano Manufacturing Technology, Guangdong 518103, R.R. China

**2. Experimental procedure**

***2.1 Synthesis of PIC-ZSH nanocomposite***

285 mg CNTs and 100 mg polyvinyl pyrrolidone (PVP K40) were added to 150 mL deionized water under ultrasonic dispersion for 60 min, forming uniform suspension. 50 mL glycol and 439.4 mg ZnAc_2_·2H_2_O were then added to the suspension and magnetically stirred for another 60 min, generating solution A. NaSnO_3_·4H_2_O (626 mg) was then dissolved into 20 mL of distilled water with ethylene glycol (in volume ratio of 3:1) under continuous stirring to form homogenous solution B. The Solution B is slowly dropped into solution A under continuous stirring. Through stirring overnight, the as-synthesized mixture was filtrated and washed by deionized water for several times to remove soluble impurities. The collected product was freeze-dried for 24 h to generate PIC-ZSH nanocomposite.

***2.2 Preparation of PIC-ZSH modulated Zn foil***

The as-synthesized PIC-ZSH nanocomposite was mixed with super conductive carbon black and polyvinylidene fluoride (PVDF) binder in a ratio of 8:1:1 by weight in an appropriate amount of N-methyl-2-pyrrolidone (NMP) solution using a planetary mixer till forming uniform slurry. Then, the slurry was casted on polished Zn foil using a film blade with a thickness of 150 µm, following a drying process at 40 °C in a vacuum oven for 48 h. The fabricated PIC-ZSH@Zn electrode was punched into discs of 16 mm in diameter as anode in Zn metal anode cell and Zn||MnO_2_ full batteries.

***2.3 Zn metal anode cell assembly***

The electrochemical performances of the cells were all estimated on CR2025-type coin cells. The Zn||Zn symmetric cell was assembled on using the as-prepared PIC-ZSH@Zn electrode as the cathode and anode separated by commercial glass fiber (*ϕ*=19 mm). The 2 mol L^-1^ (M) ZnSO_4_ solution (pH≈5) with low cost was used as electrolyte, due to its strongly coordinating SO_4_^2-^ around Zn^2+^ in ZnSO_4_ to facilitate interfacial desolvation. In the Cu||Zn asymmetrical coin cell, the cathode was replaced with Cu foil with an electrolyte containing 2 M ZnSO_4_. In addition, the coupled Zn||MnO_2_ full cells were assembled on the modified PIC-ZSH@Zn anode with commercial MnO_2_ cathode separated by commercial glass fiber, dropping the electrolyte of 2 M ZnSO_4_ with 0.1 M MnSO_4_ mixed solution.

***2.4 Materials and Devices Characterization***

X-ray diffraction (XRD) data on powders were recorded on an X-ray diffractometer (XRD, TALOS F200X Smartlab) with Cu Kα radiation at a scan rate of 5° min^-1^ in the 2θ range from 10° to 80°. X-ray photoelectron spectrometer (XPS) measurements were carried out with an ESCALAB 250XI system. The Raman spectra were outfitted with a Horiba Laser Raman spectrometer (EUROVECTOR EA3000) to determine the defective feature of materials. Scanning electron microscope (SEM) observation was performed with a Zeiss Sigma HD microscope at an accelerating voltage of 20 kV. Transmission electron microscope (TEM) was recorded on a JEM-ARM200CF NEOARM with an energy dispersive spectrometer. Confocal microscopic images were collected on a Laser Scanning Confocal Microscope (LEXT OLS4000) The 3D TOF-SIMS structures for the electrode’s reconstruction were carried out on IONTOF.

Galvanostatic charge-discharge performance of Zn||Zn symmetric batteries, Cu||Zn asymmetric batteries and Zn||MnO_2_ full batteries under different current rates were carried out on a multichannel battery test system (BTS-5 V 20 mA, Neware, Shenzhen), which was commenced after an 8 h resting period. The low-temperature environment is provided by a low-temperature test chamber (T-107-C, TOTA Shanghai) with rated temperature of -40~130 ^o^C. Before conducting low-temperature performance test, it is necessary to place the assembled batteries in the chamber and stabilize at target temperature for 6 h. Corrosion curves (Tafel) were performed at room temperature in a three-electrode system using a Zn plate (1 cm^-2^) as working electrode, Pt plate as auxiliary electrode and calomel electrode (Saturated KCl) as reference electrode, respectively, at a scan rate of 1 mV s^-1^ with a 2 M ZnSO_4_ solution. The cyclic voltammetry (CV) and electrochemical impedance spectroscopy (EIS) data were performed on an electrochemical workstation (CHI660E, Shanghai) using a two electrodes system. The EIS plots were performed within the frequency range of 10^-2^-10^5^ Hz. The CV test was recorded at a constant sweeping speed of 0.1 mV s^-1^ or shifty sweeping speeds from 0.1 to 1 mV s^-1^.

***2.5 COMSOL Multiphysics simulation***

COMSOL Multiphysics 6.2 was used to simulate the current density distributions and ionic concentration. The two-dimensional (2D) transient model and tertiary Nernst-Planck equation (1) were chosen to simulate ionic concentration distribution. The number of participating electrons is set to 2. The initial concentration of Zn^2+^ was 1 mol L^-1^. According to Fick's law and Poisson's equation, the ion diffusion was affected by the concentration gradient of ions and the distribution of electric field. During operation, the battery follows the principle of electronic charge conservation and mass conservation.

$J_{i}\left( x \right)=-D_{i}\frac{\partial C_{i}\left( x \right)}{\partial x}-\frac{z_{iF}}{RT}D_{i}C_{i}\frac{\partial\Phi\left( x \right)}{\partial x}+C_{i}v(x)$ (1)

J_i_(x) is the flow rate of substance i at the distance x from the electrode surface, mol s^-1^ cm^-2^; D_i_ is the diffusion coefficient, cm^-2^ s^-1^; $\frac{\partial C_{i}(x)}{\partial x}$is the concentration gradient at x; $\frac{\partial\Phi\left( x \right)}{\partial x}$is the potential gradient; Z_i_ and C_i_ are the charge and concentration of substance i, respectively, the three items on the right of the formula represent the contribution of diffusion, migration and convection to the flow.


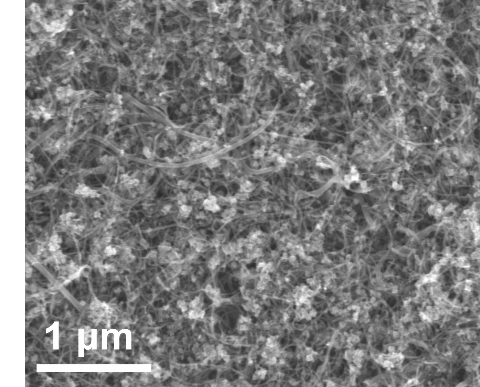


**Figure S1** SEM image of the PIC-ZSH nanocomposite.


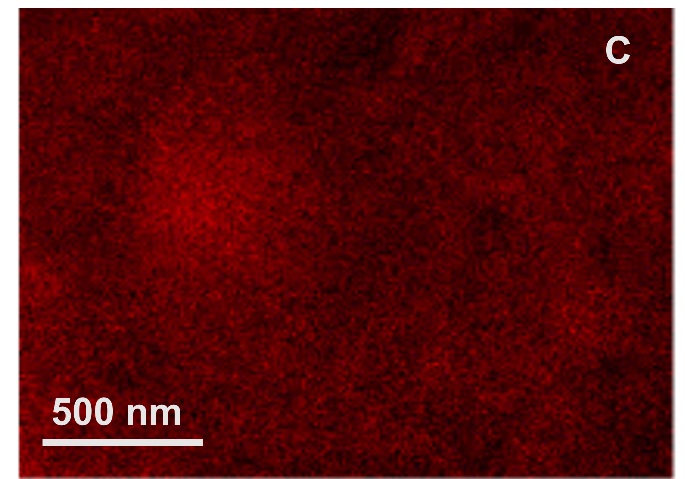


**Figure S2** EDS mapping of C in the PIC-ZSH nanocomposite.


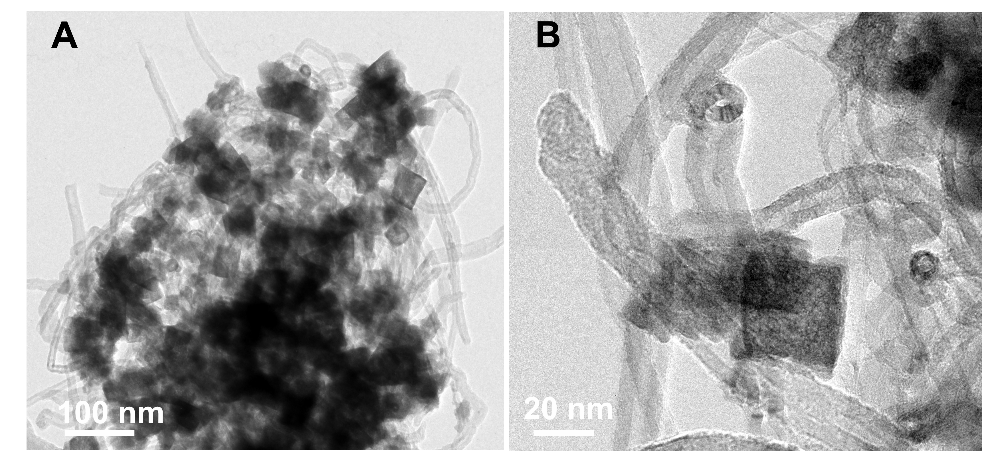


**Figure S3** TEM images of the PIC-ZSH nanocomposite.





**Figure S4** Raman spectrum of the PIC-ZSH.





**Figure S5** XPS survey spectra of the PIC-ZSH.


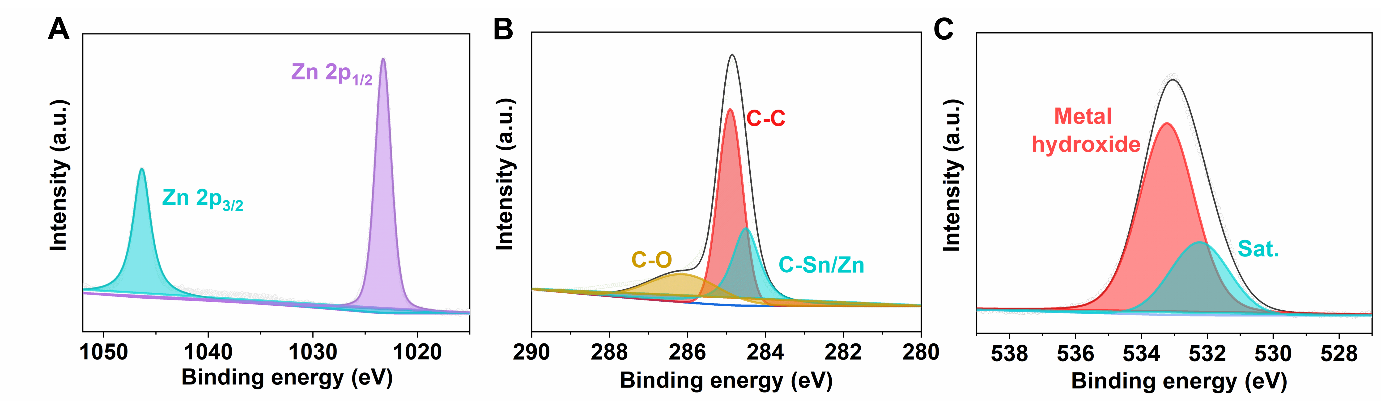


**Figure S6** High-resolution XPS spectra of (A) Zn 2p, (B) C1s and (C) O1s regions of the PIC-ZSH nanocomposite.


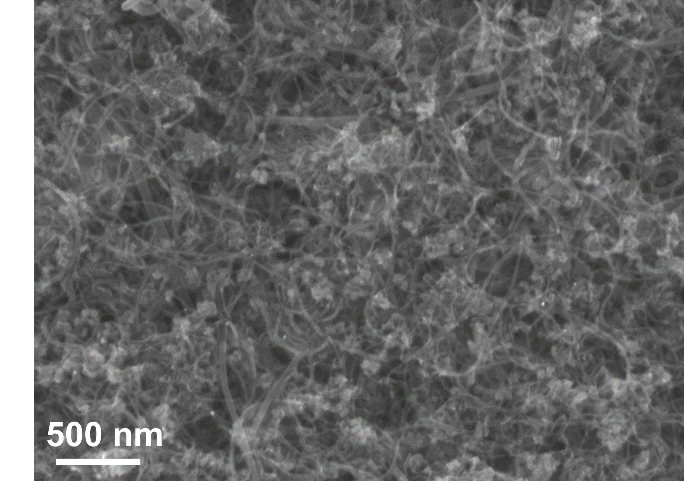


**Figure S7** SEM image of the PIC-ZSH modulated Zn.


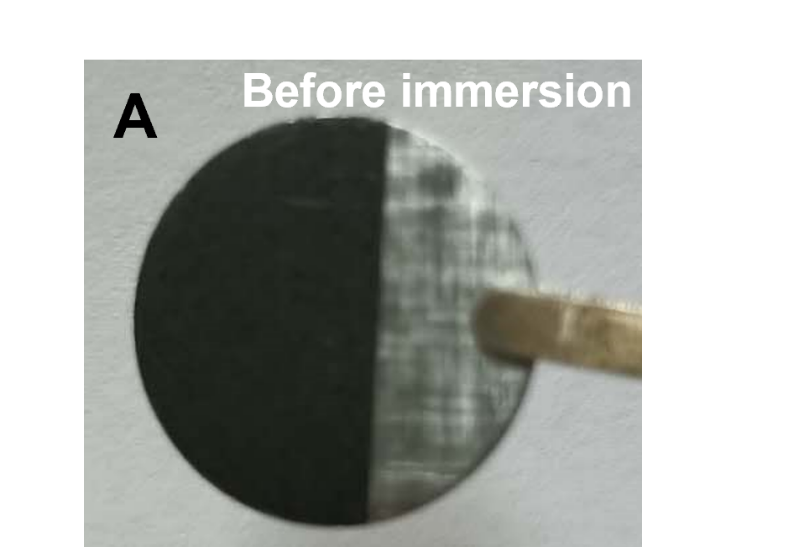


**Figure S8** Optical image of the partially coated PIC-ZSH@Zn electrode before immersion in the electrolyte.


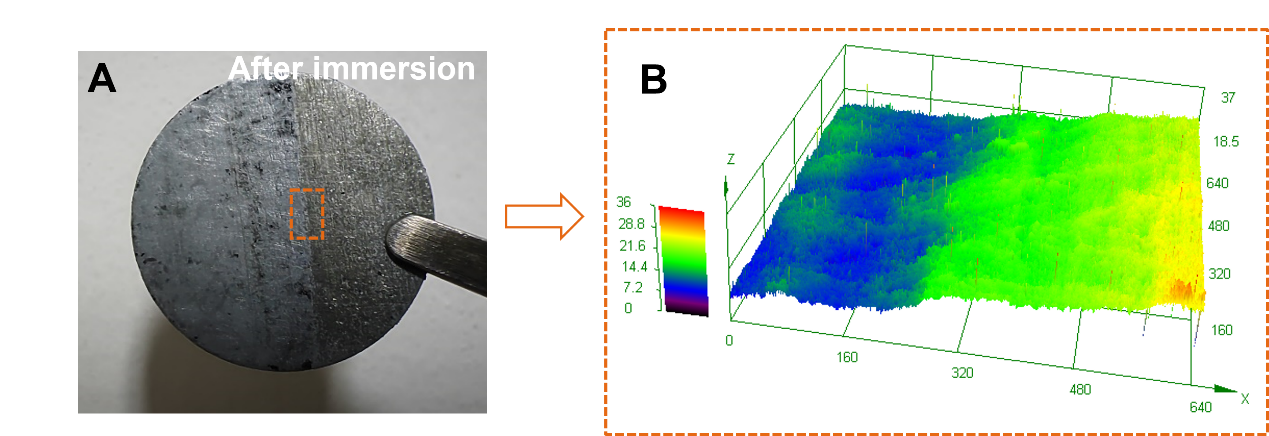


**Figure S9** (A) Optical image of the immersed PIC-ZSH@Zn electrode after removing the upper modulation layer and (B) the corresponding confocal microscopic 3D morphology.





**Figure S10** Raman spectra of electrolyte on bare Zn and PIC-ZSH@Zn.


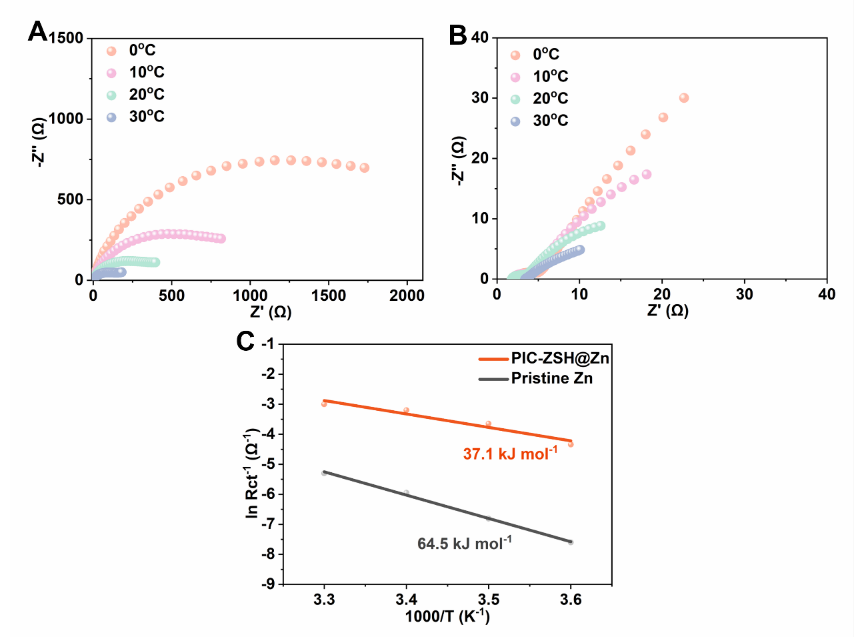


**Figure S11** Electrochemical impedance spectroscopy (EIS) plots of (a) PIC-ZSH@Zn and (b) pristine Zn at different temperatures ranging from 0°C to 30°C; (c) Desolvation activation energy of PIC-ZSH@Zn and pristine Zn fitted to the Arrhenius equation.


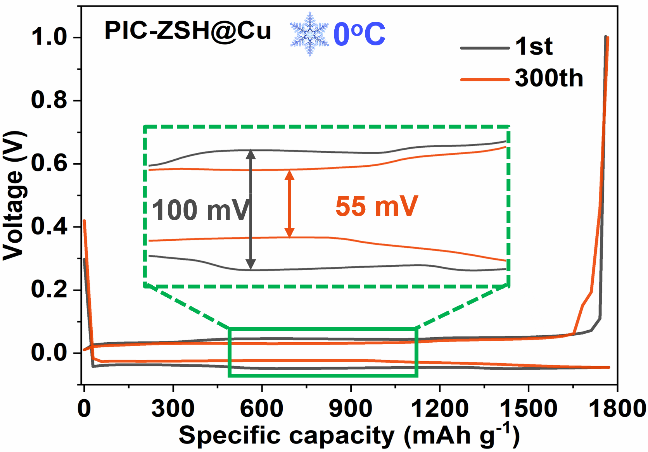


**Figure S12** Voltage-capacity profiles comparison of the PIC-ZSH@Cu electrodes in the 1^st^ and the 300^th^ cycle planting 1 mA h cm^-2^ capacity of Zn at 1 mA cm^-2^ (the inset comparison curve of the overpotential on different cycles in asymmetric cell).





**Figure S13** EIS spectra for symmetric cells based on PIC-ZSH@Zn and pristine Zn electrodes.


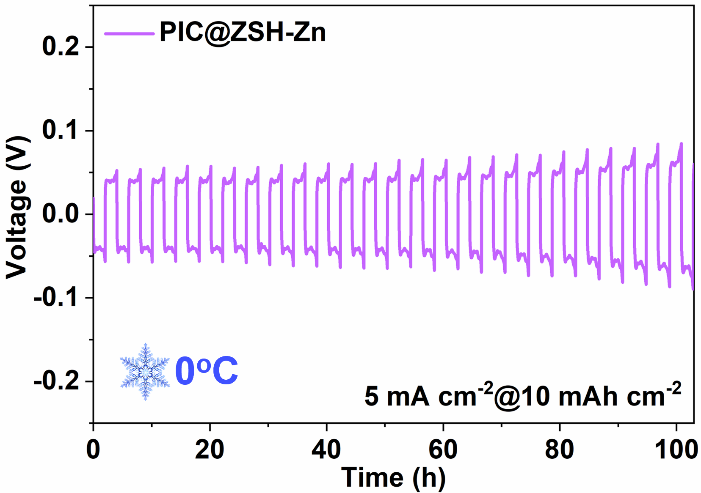


**Figure S14** Galvanostatic Zn stripping/plating behavior at 5 mA cm^-2^ with 10 mAh cm^-2^ under the low temperature of 0 °C.


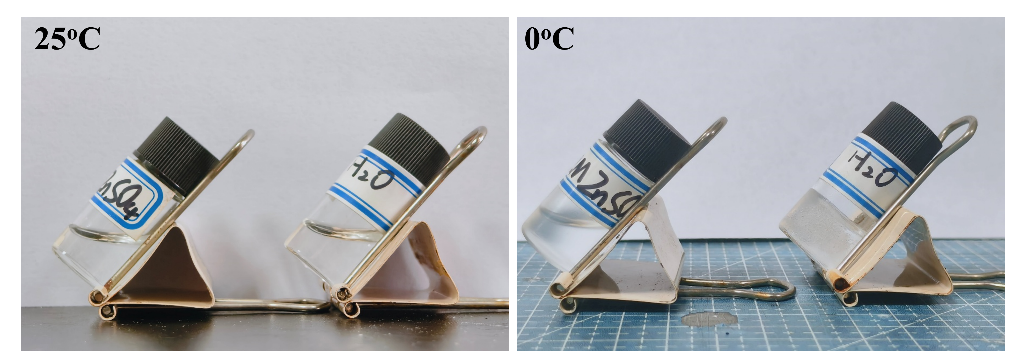


**Figure R15** The state of 2M ZnSO_4_ electrolyte under 25 and 0 ℃.


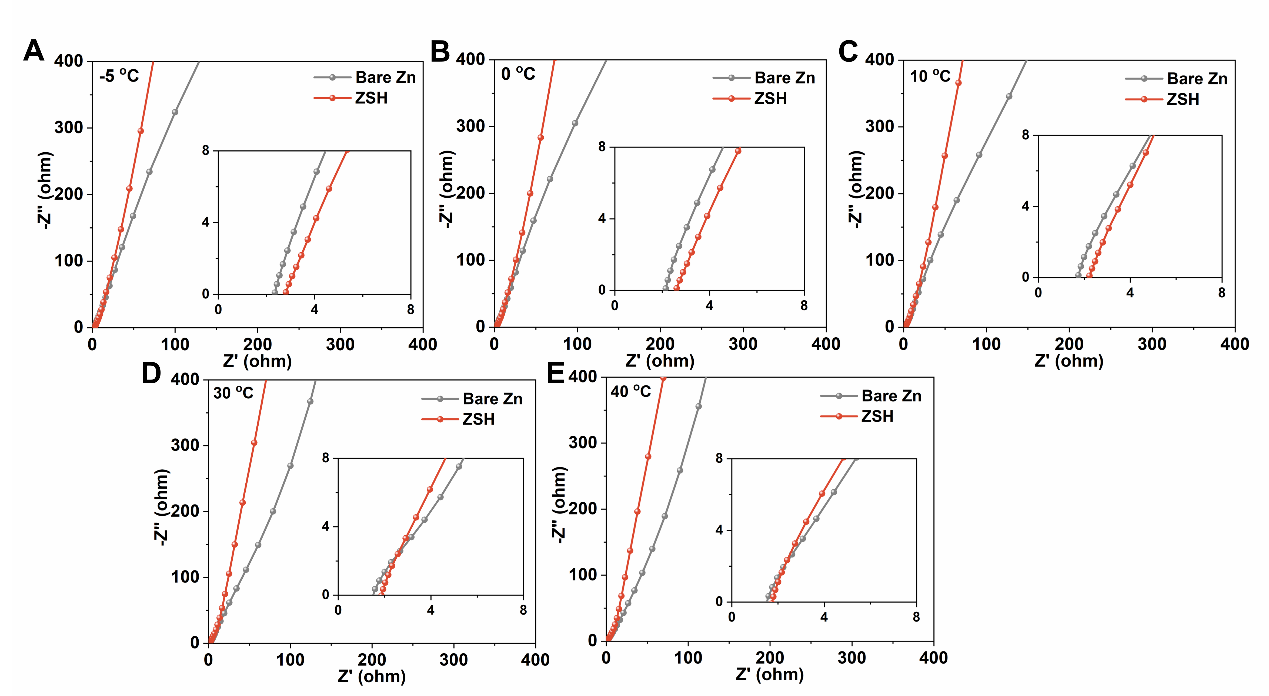


**Figure S16** Ionic conductivity tests under different temperature.


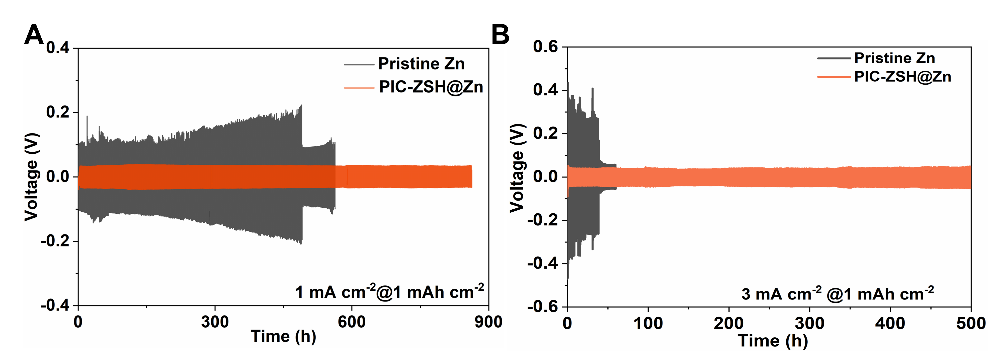


**Figure S17** Galvanostatic Zn stripping/plating behavior (A) at 1 mA cm^-2^ with 1 mAh cm^-2^ and (B) at 3 mA cm^-2^ with 1 mAh cm^-2^ under room temperature.





**Figure S18** Galvanostatic Zn stripping/plating from 0.2 to 10 mA cm^-2^ at room temperature.


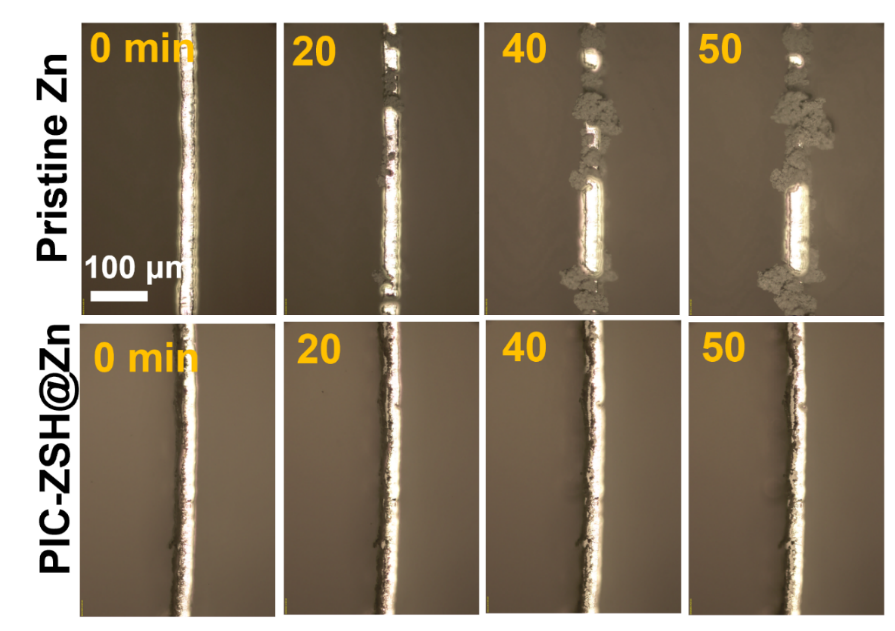


**Figure S19** In situ optical microscopy observation of Zn plating on pristine Zn and PIC-ZSH@Zn anodes at 5 mA cm^-2^.


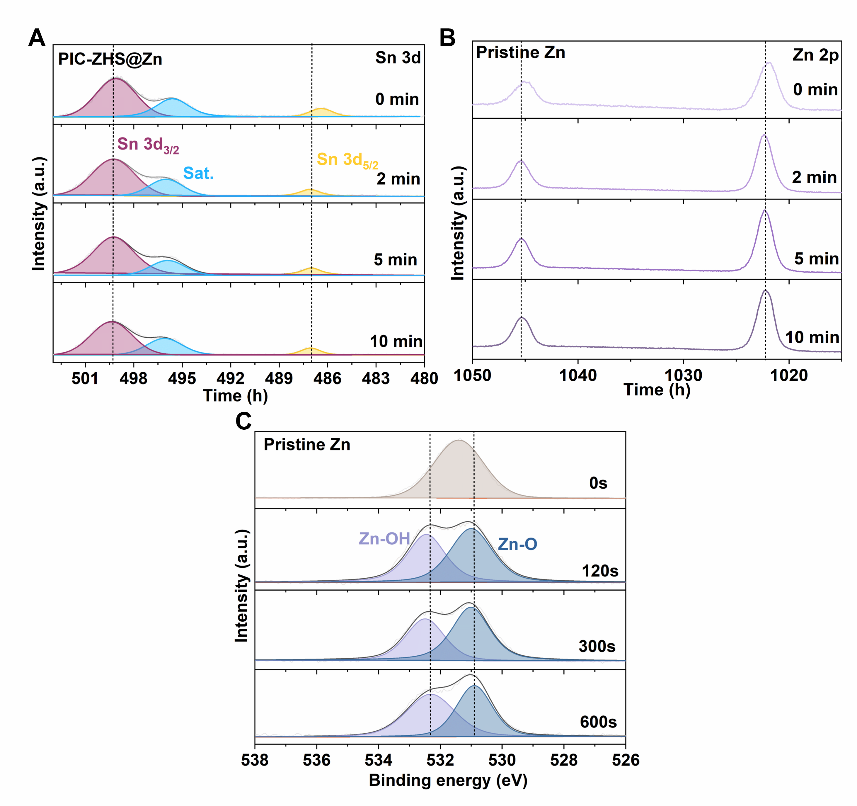


**Figure S20** XPS depth profiles of SEI formed on the Zn/electrolytes interface after cycling for 30 h at 0 °C, including high-resolution (A) Sn 3d spectra for PIC-ZSH@Zn electrode, (B) Zn 2p and (C) O 1s spectra for pristine Zn.


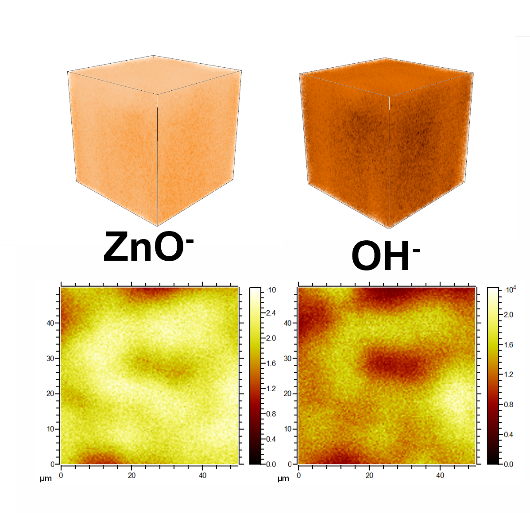


**Figure S21** Interfacial 3D and 2D reconstruction of species within interfacial SEI on pristine Zn.


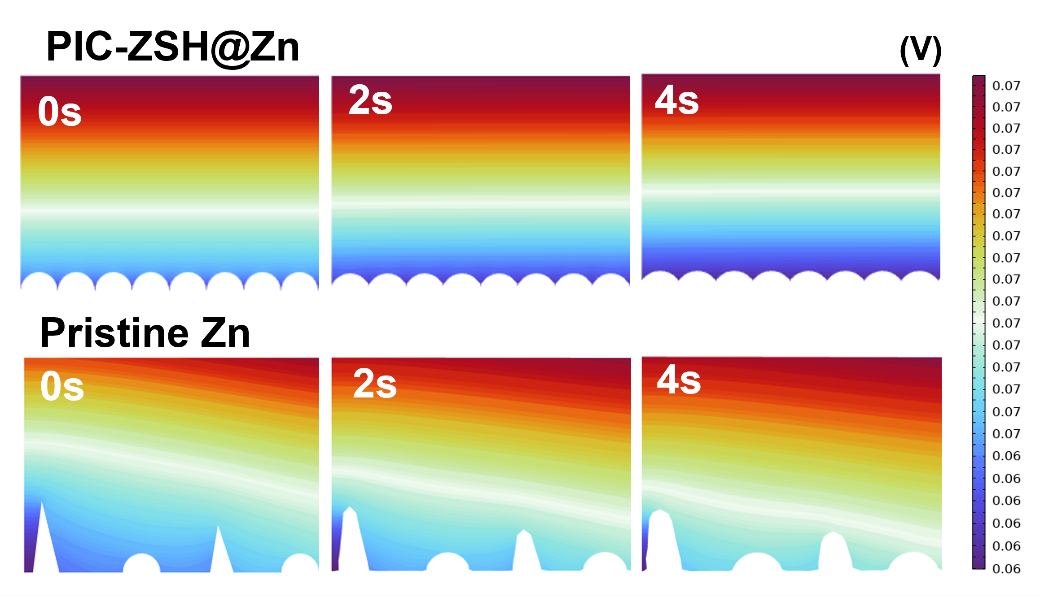


**Figure S22** COMSOL simulation of the voltage distributions on the PIC-ZSH@Zn and pristine Zn electrodes surface from a side view, respectively.





**Figure S23** Capacity-potential curves of PIC-ZSH@Zn||MnO_2_ full cell at 1A g^-1^ under shifting temperature.


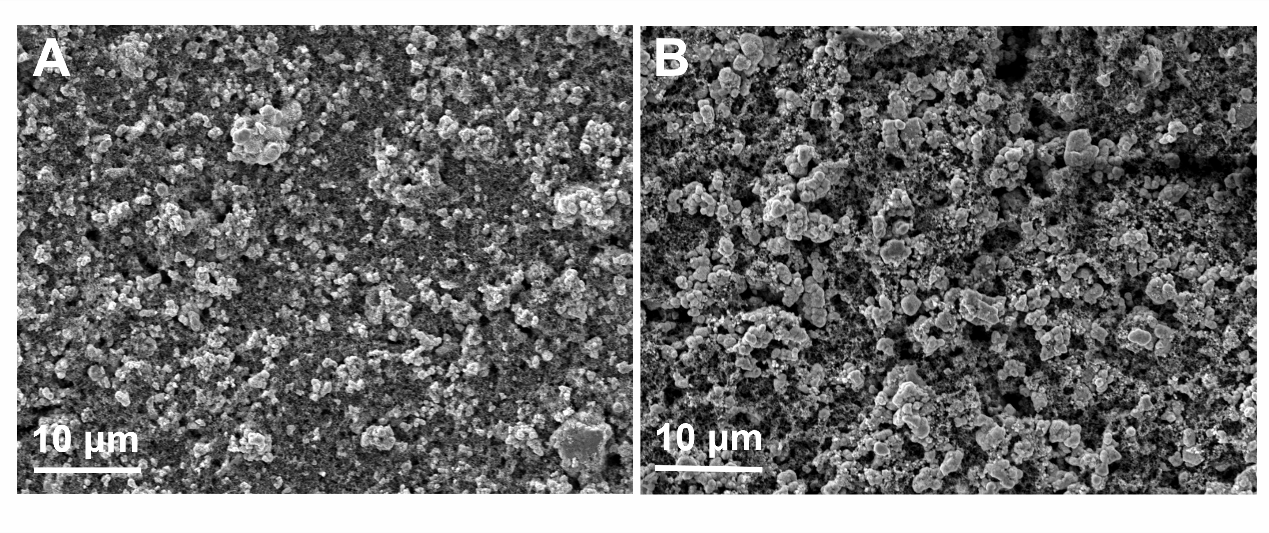


**Figure S24** SEM images of MnO_2_ electrode (A) before and (B) after cycling.





**Figure S25** XRD pattern of MnO_2_ electrode after cycling.

**Table S1** The temperature-dependent ionic conductivity of the PIC-ZSH layer.

| Temperature (°C) | Ionic conductivity (mS cm^-1^) |
| --- | --- |
| -5 | 7.4 |
| 0 | 7.7 |
| 10 | 7.9 |
| 30 | 10.9 |
| 40 | 17.7 |

**Table S2.** A comparison of symmetrical battery performance with current reports

| **Electrode** | **Current density**  **(mA cm^-2^)** | **Areal capacity**  **(mAh cm^-2^)** | **Lifespan**  **(h)** | **Test**  **Temperature (^o^C)** | **Ref.** |
| --- | --- | --- | --- | --- | --- |
| **PIC-ZSH@Zn** | **1** | **1** | **1300** | **0** | **This work** |
|  | **10** | **2** | **800** | **0** |  |
| ɑ-BTO/PVT@Zn | 5 | 1 | 900 | 30 | ^[1]^ |
| Zn-N_3py+1pr_-C@Zn | 1 | 1.5 | 750 | 30 | ^[2]^ |
| Bi-N_4_/C@Zn | 1 | 1 | 425 | -30 | ^[3]^ |
| BN@Zn | 1 | 1 | 300 | -10 | ^[4]^ |
| Nb_2_O_5_ modified Zn | 1 | 0.5 | 1000 | 30 | ^[5]^ |
| t-KTN modified Zn | 1 | 1 | 1200 | 30 | ^[6]^ |
| BaTiO_3_ modified Zn | 10 | 3 | 600 | 30 | ^[7]^ |
| 3D ZnF_2_ | 1 | 1 | 800 | 30 | ^[8]^ |

**Reference**

[1] Q. Zong, B. Lv, C. Liu, Y. Yu, Q. Kang, D. Li, Z. Zhu, D. Tao, J. Zhang, J. Wang, Q. Zhang, G. Cao, *ACS Energy Lett.* **2023**, 8, 2886.

[2] Z. Yang, F. Lai, Q. Mao, C. Liu, R. Wang, Z. Lu, T. Zhang, X. Liu, *Adv. Mater.* **2024**, 36, 2311637.

[3] S. Chen, J. Chen, X. Liao, Y. Li, W. Wang, R. Huang, T. Zhao, S. Yan, Z. Yan, F. Cheng, H. Wang, *ACS Energy Letters* **2022**, 7, 4028.

[4] H. Jia, M. Qiu, C. Tang, H. Liu, S. Fu, X. Zhang, *EcoMat* **2022**, 4, e12190.

[5] S. So, Y. N. Ahn, J. Ko, I. T. Kim, J. Hur, *Energy. Storage. Mater.* **2022**, 52, 40.

[6] T. Chen, F. Huang, Y. Wang, Y. Yang, H. Tian, J. M. Xue, *Adv Sci (Weinh)* **2022**, 9, e2105980.

[7] S. Zhou, X. Meng, C. Fu, D. Xu, J. Li, Q. He, S. Lin, S. Liang, Z. Chang, A. Pan, *Small* **2023**, 19, e2303457.

[8] Y. Yang, C. Liu, Z. Lv, H. Yang, Y. Zhang, M. Ye, L. Chen, J. Zhao, C. C. Li, *Adv. Mater.* **2021**, 33, 2007388.
